# Supplementary material for: Transposable elements are regulated by context-specific patterns of chromatin marks in mouse embryonic stem cells
Source: Nat Commun. 2019 Jan 3;10:34. doi: 10.1038/s41467-018-08006-y (PMC6318327; doi:10.1038/s41467-018-08006-y)
Supplement: Supplementary file 1 — Supplementary Information [file 41467_2018_8006_MOESM1_ESM.pdf]

Supplementary Information

Transposable elements are regulated by context-specific patterns of chromatin marks in mouse embryonic stem cells

He et al.,

Supplementary Discussion

In this study, many TEs appear to lack any chromatin mark. Whilst many TEs may lack any chromatin modifications, many may be marked by histone modifications that have not yet been experimentally surveyed. We analyzed 32 chromatin modifications in our study, but mass spectrometry experiments revealed at least 100 distinct histone modifications[1,2], not including many understudied modifications, such as crotonylation or propionylation, and larger moieties such as ubiquitination and sumoylation[1,3-5]. Several of these epigenetic marks have already been associated with gene expression[5], and it seems likely they will also be involved in regulating TEs.

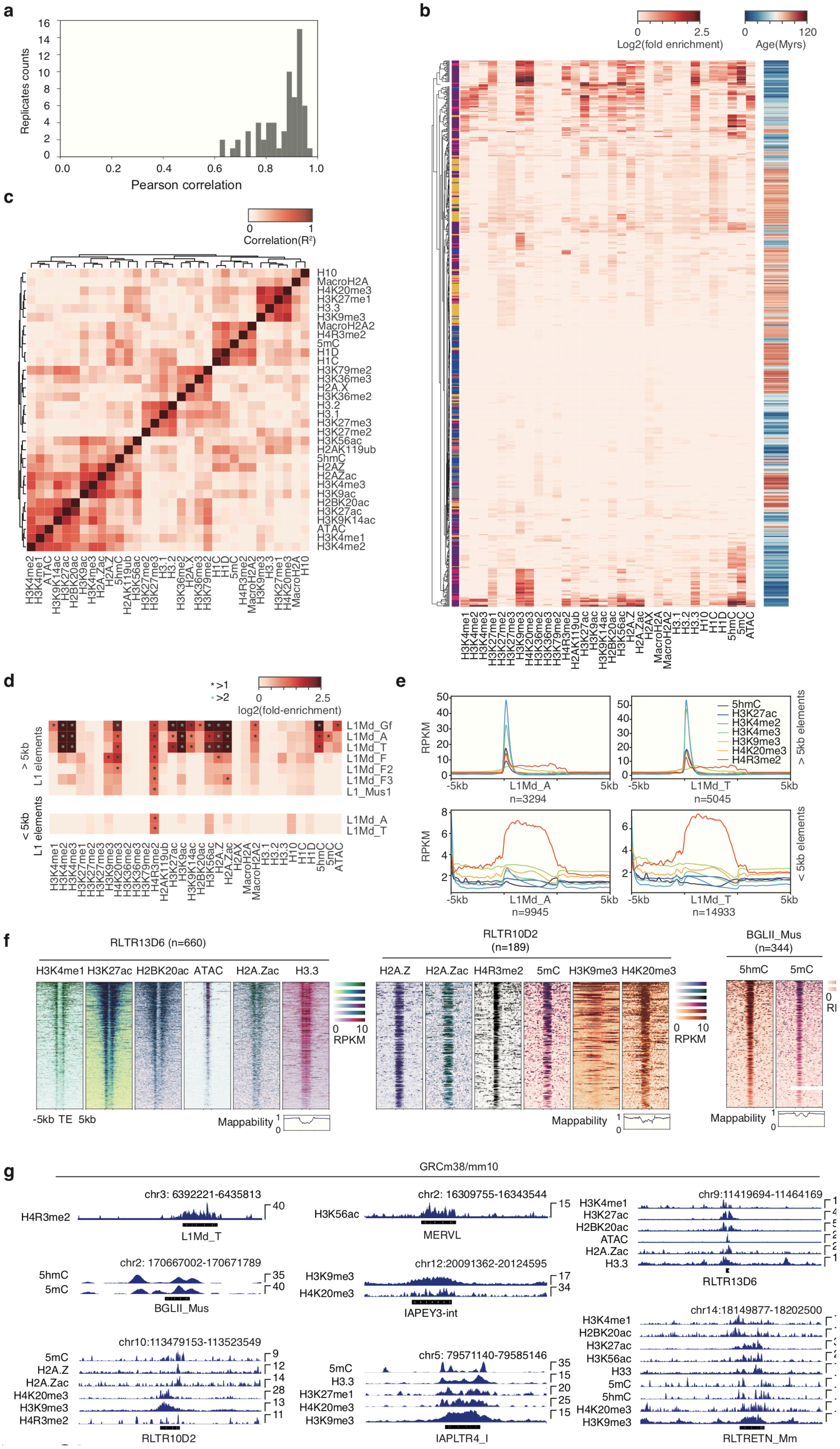
a
Replicates counts
Pearson correlation
b
Log2(fold enrichment)
Age(Myrs)
c
Correlation(R²)
H10
MacroH2A
H4K20me3
H3K27me1
H3.3
H3K9me3
MacroH2A2
H4R3me2
5mC
H1D
H1C
H3K79me2
H3K36me3
H2A.X
H3K36me2
H3.2
H3.1
H3K27me3
H3K27me2
H3K56ac
H2AK119ub
5hmC
H2AZ
H2AZac
H3K4me3
H3K9ac
H2BK20ac
H3K27ac
H3K9K14ac
ATAC
H3K4me1
H3K4me2
d
*>1
*>2
log2(fold-enrichment)
> 5kb L1 elements
< 5kb L1 elements
L1Md_Gf
L1Md_A
L1Md_T
L1Md_F
L1Md_F2
L1Md_F3
L1_Mus1
e
RPKM
5hmC
H3K27ac
H3K4me2
H3K4me3
H3K9me3
H4K20me3
H4R3me2
> 5kb elements
< 5kb elements
-5kb
5kb
L1Md_A
n=3294
L1Md_T
n=5045
n=9945
n=14933
f
RLTR13D6 (n=660)
H3K4me1
H3K27ac
H2BK20ac
ATAC
H2A.Zac
H3.3
RPKM
-5kb TE 5kb
Mappability
RLTR10D2 (n=189)
H2A.Z
H2A.Zac
H4R3me2
5mC
H3K9me3
H4K20me3
BGLII_Mus (n=344)
5hmC
5mC
g
GRCm38/mm10
chr3: 6392221-6435813
H4R3me2
L1Md_T
chr2: 16309755-16343544
H3K56ac
MERVL
chr9:11419694-11464169
H3K4me1
H3K27ac
H2BK20ac
ATAC
H2A.Zac
H3.3
RLTR13D6
chr2: 170667002-170671789
5hmC
5mC
BGLII_Mus
chr12:20091362-20124595
H3K9me3
H4K20me3
IAPEY3-int
chr14:18149877-18202500
H3K4me1
H2BK20ac
H3K27ac
H3K56ac
H33
5mC
5hmC
H4K20me3
H3K9me3
RLTRETN_Mm
chr10:113479153-113523549
5mC
H2A.Z
H2A.Zac
H4K20me3
H3K9me3
H4R3me2
RLTR10D2
chr5: 79571140-79585146
5mC
H3.3
H3K27me1
H4K20me3
H3K9me3
IAPLTR4_I

Supplementary Figure 1. TEs are marked by specific chromatin marks. (a) Quality control for ChIP-seq/epigenetic-seq data. Data was downloaded from GEO/SRA[6] and the FASTQ files were reanalyzed. Pearson correlation was measured between biological replicates of the same type, by taking the RPKM (reads per kilobase per million reads) for all TE types. ChIP/epigenetic-seq samples with a correlation less than 0.6 to any two other biological replicates were deleted from the analysis. See **Supplementary Data 1** for details of the samples used in this study. (b) Heatmap of all TE types (rows), against all chromatin marks (columns), hierarchically clustered, showing the fold-enrichment of the chromatin mark. The estimated age of the TE type is indicated on the right, in millions of years (Myrs). The full table is in **Supplementary Data 2**. (c) Pair-wise R^2 correlation plot for the patterns of chromatin mark for all TE types. (d) Heatmap showing the fold-enrichment over a random background of the chromatin patterns for long (>5 kb) and short (<5 kb) LINE elements. (e) Pileup tag density plots for a selection of chromatin marks over long (>5 kb) and short (<5 kb) L1Md_A and L1Md_T elements. The LINEs are aligned by their 5' ends, and the flanking 5 kb regions are shown. (f) Selected pileup heatmaps for the indicated TEs, and the indicated chromatin marks. The number of TEs in each heatmap is indicated (n=). Average sequence read mappability is indicated below the right most heatmap for each TE. These TE types are shown as they have overall good mappability (>0.6) across the TE. The TEs were scaled to the same size, and the flanking 5kb regions are shown. (g) Example genome views of chromatin marks for the TEs: LINE1 (L1Md_T) ERVKs (BGLII_Mus, IAPLTR4_I, RLTR13G, RLTRETN_Mm, RLTR10D2) and ERVL (MERVL). The range of the plots goes from 0, up to the values indicated on the right side of each histogram. Genomic locations are from the mm10 mouse assembly. Care should be taken in interpreting genomic views due to uncertainty in mapping reads to specific TE copies, this applies to all genome views in the manuscript.

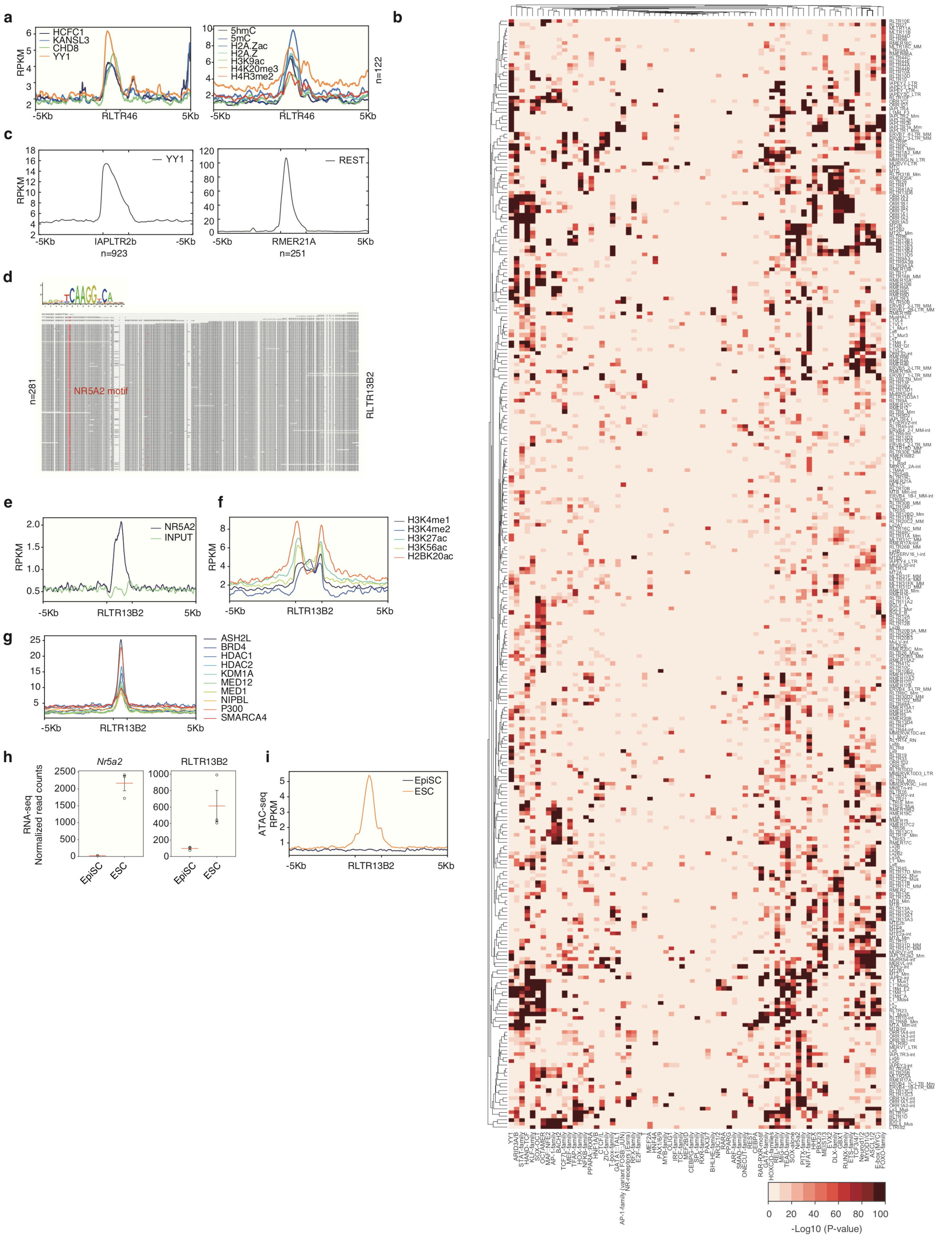

Supplementary Figure 2. TEs recruit a wide-range of chromatin modifiers. (a) Sequence tag pileups for CMs (left) and chromatin marks (right) on RLTR46. RLTR46 was scaled to the same size, and the flanking 5kb regions are shown. (b) Motif enrichment within the TEs that have enriched CMs. Motifs were detected with MEME[7]. (c) Sequence tag pileups for YY1 (left)

and REST (right) at the indicated TE types. The number of copies of the TE are indicated (n=). (d) Alignment of all of the genomic copies of the RLTR13B2, showing the location of the NR5A2 motif in red. The consensus NR5A2 sequence logo is indicated at the top of the TE. The number of copies of the TE are indicated (n=). (e) Sequence density pileups for NR5A2 ChIP-seq data, and its matching Input control. NR5A2 ChIP-seq data was from GSE19019[8]. (f, g) ChIP-seq pileup for the indicated chromatin marks (panel f) and CMs (panel g), displayed as in **panel e**. (h) Expression from RNA-seq data for ESCs and EpiSCs for *Nr5a2* (left) and RLTR13B2 TE (right). RNA-seq data was taken from GSE99491[9]. The red line indicates the mean and the error bars the standard error of the mean of the three biological replicates. Source data are provided as a **Source Data** file. (i) Chromatin accessibility (ATAC-seq data) for RLTR13B2 TE in ESCs and EpiSCs. ATAC-seq data was taken from GSE101074[10].

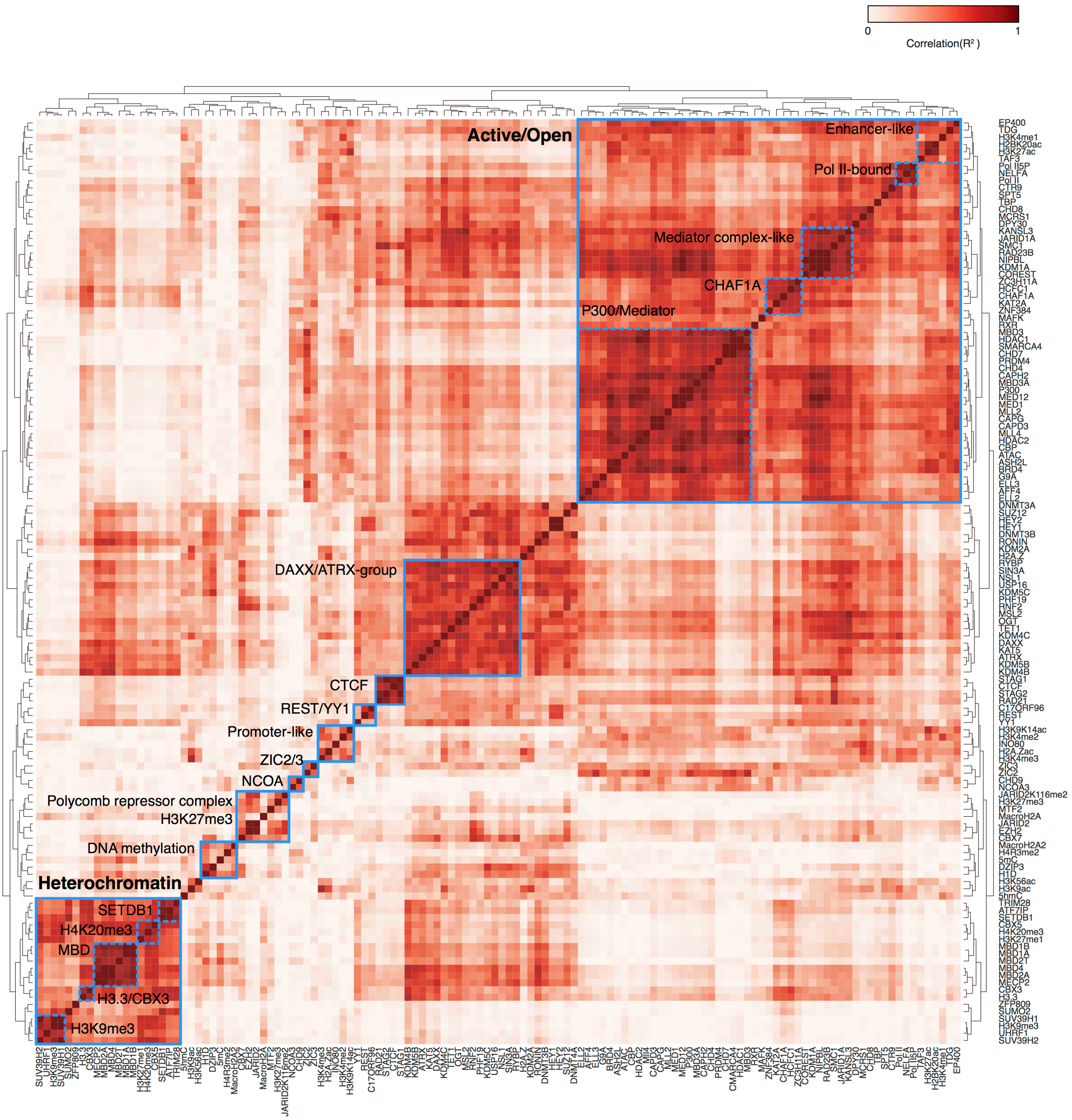

Supplementary Figure 3. Pair-wise correlation matrix for all chromatin modifiers and chromatin modifications. Pair-wise R^2 correlation plot for the patterns of chromatin mark for all TEs and CMs analyzed in this study. Selected groups are labelled, including the two super-groups we designate Active/Open and Heterochromatin. Within the supergroups are several subgroups that are annotated based on the major regulators within each sub cluster. The Active/Open supergroup contains a P300/Mediator subgroup (P300, MED1/12), CHAF1A, Mediator-like (NIPBL, SMC1), Pol II-bound (Pol II, NELFA), and Enhancer-like (EP400, H3K4me1). The Heterochromatin supergroup contains a SETDB1 cluster (SETDB1, TRIM28), a H4K20me3 cluster (H4K20me3, H3K2me1, CBX5), an MBD cluster (MBD1A/1B/2A/2T/4), a H3.3 cluster, and finally a H3K9me3 cluster (H3K9me3, SUV39H1/2). In addition, there are several smaller subgroups. These include DNA methylation (5mC, H1D, DZIP3), Polycomb-repressor complex (H3K27me3, EZH2, JARID2), NCOA, ZIC2/3, promoter-like (H3K4me3, H3(K9/K14)ac), REST/YY1, and CTCF. Another major group was a cluster containing DAXX/ATRX.

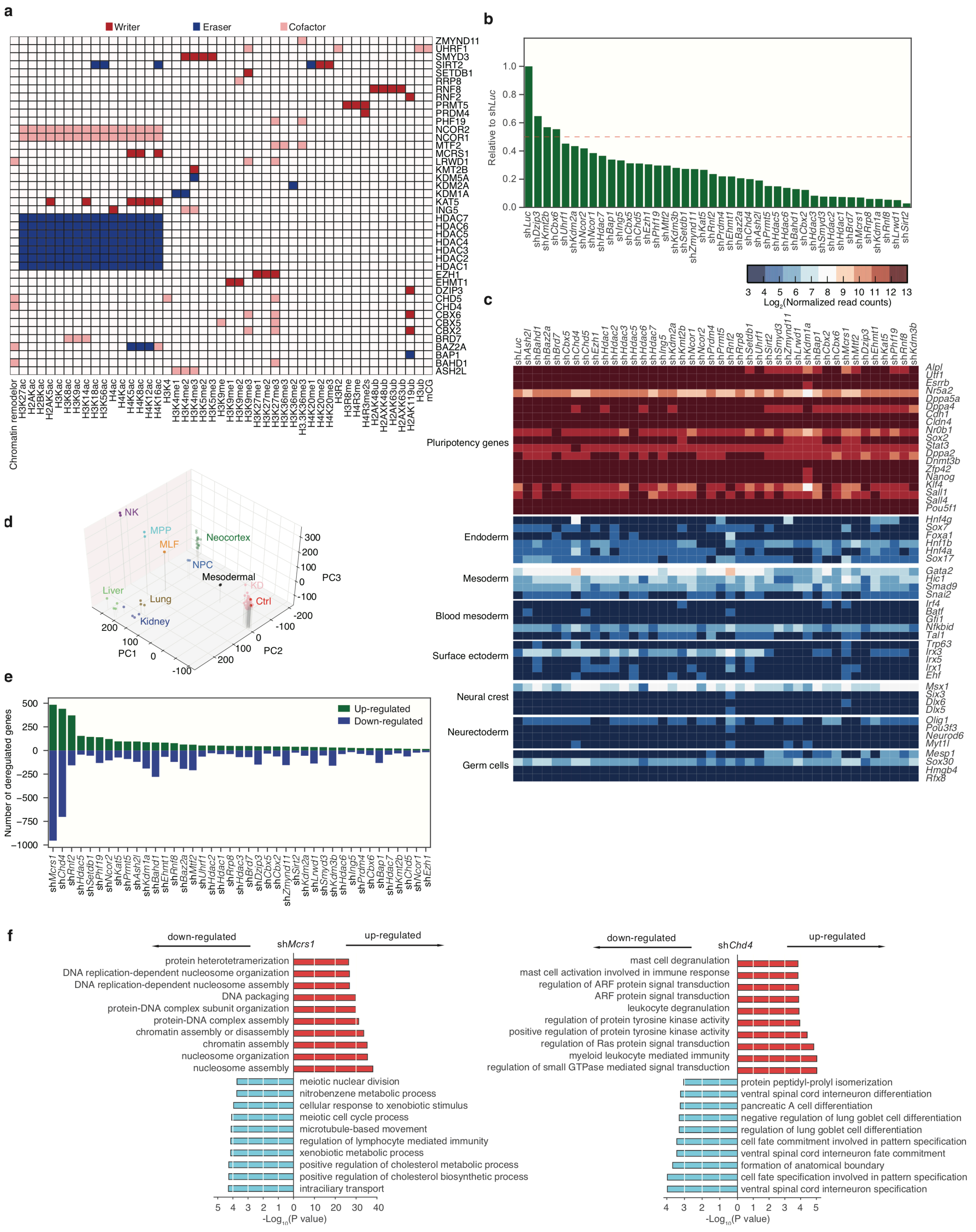

Supplementary Figure 4. Knockdown of CMs does not drastically alter cell state. (a) Table of known chromatin targets of the CMs used in this study. Red boxes indicate the indicated CM is known to catalyze (directly or indirectly) that chromatin mark, blue indicates the CM can remove that mark, and pink indicates that the CM is known to bind to or read the mark. Data was taken from the EpiFactors database[11]. (b) Knockdown efficiency relative to sh*Luc* for the

indicated knockdowns. Data was derived from the RNA-seq data. (c) Gene expression heatmap for a selection of pluripotency-related genes and a selection of differentiation-associated genes for the germ lineages: endoderm, mesoderm, blood mesoderm, surface ectoderm, neural crest, neurectoderm and germ cells. Lineage-specific genes were taken from[12]. (d) Principal component analysis of the shRNA knockdowns (KD, pink) and control sh*Luc* knockdowns (Ctrl, red), compared to gene expression in a range of somatic cell types. Raw data was downloaded from GSE60101, GSE20851, GSE41637, GSE38805 and GSE47948 (See **Supplementary Data 1**) and reprocessed using the same RNA-seq pipeline as the other RNA-seq data in this study. (e) Numbers of significantly differentially regulated genes in the indicated knockdowns. A gene was considered deregulated by DESeq2 if the P value<0.05 (Benjamini-Hochberg corrected) and the absolute fold-change was greater than 2. (f) Gene ontology analysis of the significantly up and down regulated genes in the *Mrcs1* knockdown (left) and the *Chd4* knockdown (right).

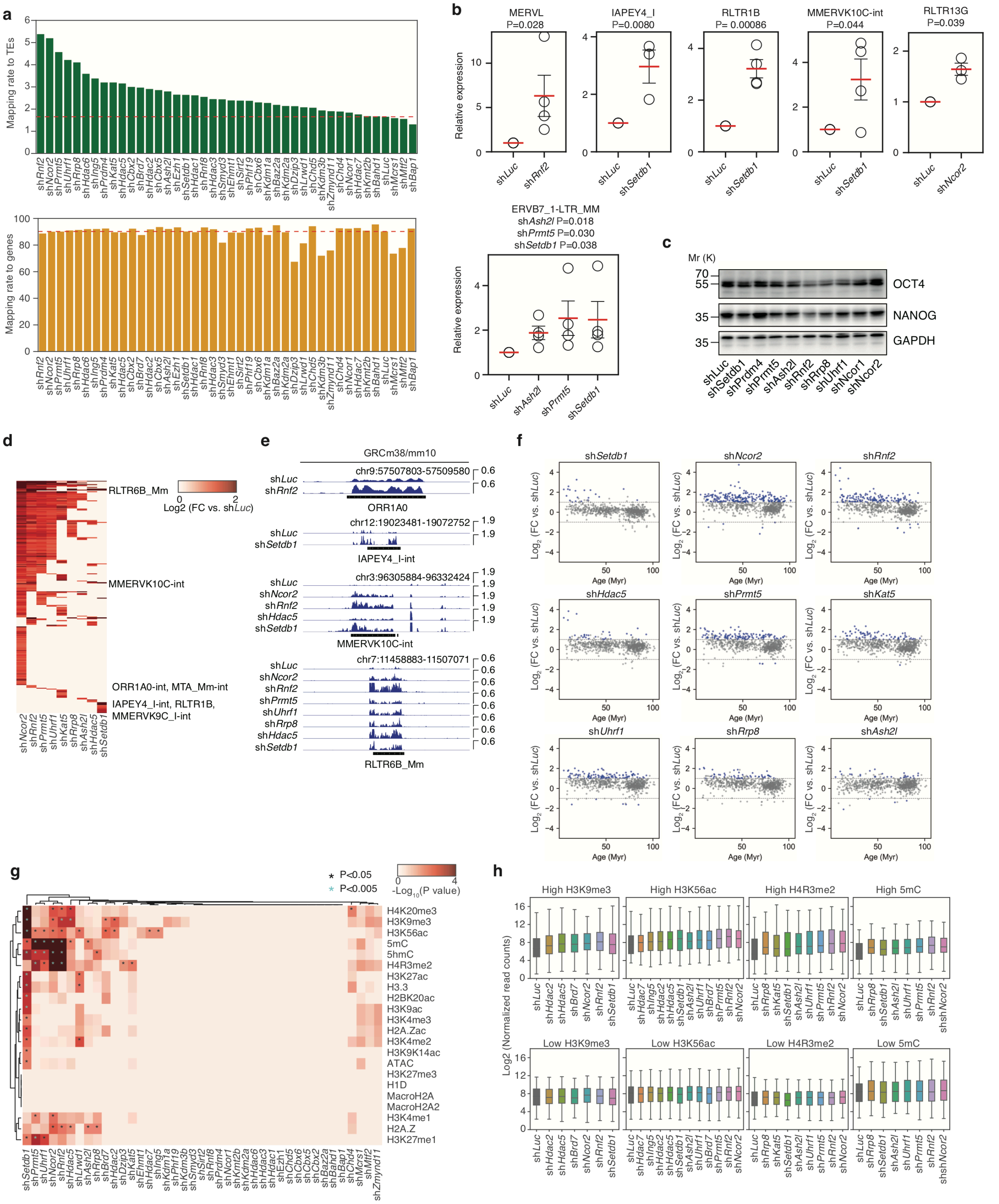

Supplementary Figure 5. RNA-seq mapping to TEs, validation and association with chromatin marks. (a) Percent of RNA-seq reads mapping to TEs (top) and to genes (bottom). The red line indicates the % of reads mapping to the sh*Luc* control. Each bar is the average of the replicates. (b) RT-qPCR validation of selected TEs in the indicated shRNA knockdowns, relative to sh*Luc*. P values are from a one-tailed Student's t-test. The circles are the mean of 3 technical replicates, the red bar is the mean of the 3 biological replicates, and the error bars are the standard error of the mean. Primers used are described in **Supplementary Data 7**.

Source data are provided as a **Source Data** file. (c) Western blot for the pluripotency proteins OCT4, NANOG and GAPDH, for the indicated shRNA knockdowns. Molecular weight is indicated as Mr (K). This experiment was performed twice with similar results. (d) Fold change (relative to sh*Luc*) of all TEs significantly differentially regulated in the indicated knockdowns. (e) Example genome views of RNA-seq data for the selected TEs in the indicated knockdowns. (f) Scatter plot showing the relationship between TE fold-change deregulation, and TE age (in millions of years/Myr) in the indicated shRNA knockdowns. Significantly differentially regulated TEs are indicated in blue. (g) Heatmap showing the significant associations between chromatin marks and the TEs deregulated in the indicated shRNA knockdown. P value is from a Fisher's exact test, * indicates P value <0.05. (h), Boxplots of the change in expression for all TEs with either high or low levels, for a selection of chromatin marks and shRNA knockdowns from **panel g**. High was defined as all those TE types with fold enrichment >2 for the indicated chromatin mark. A background of low sites was selected by ranking all TE types from top to bottom by fold enrichment, and taking the TEs from the bottom of the list until it was the same size as the number of TEs in the high list. For the boxplots, the midline indicates the median, boxes indicate the upper and lower quartiles and the whiskers indicate 1.5*interquartile range.

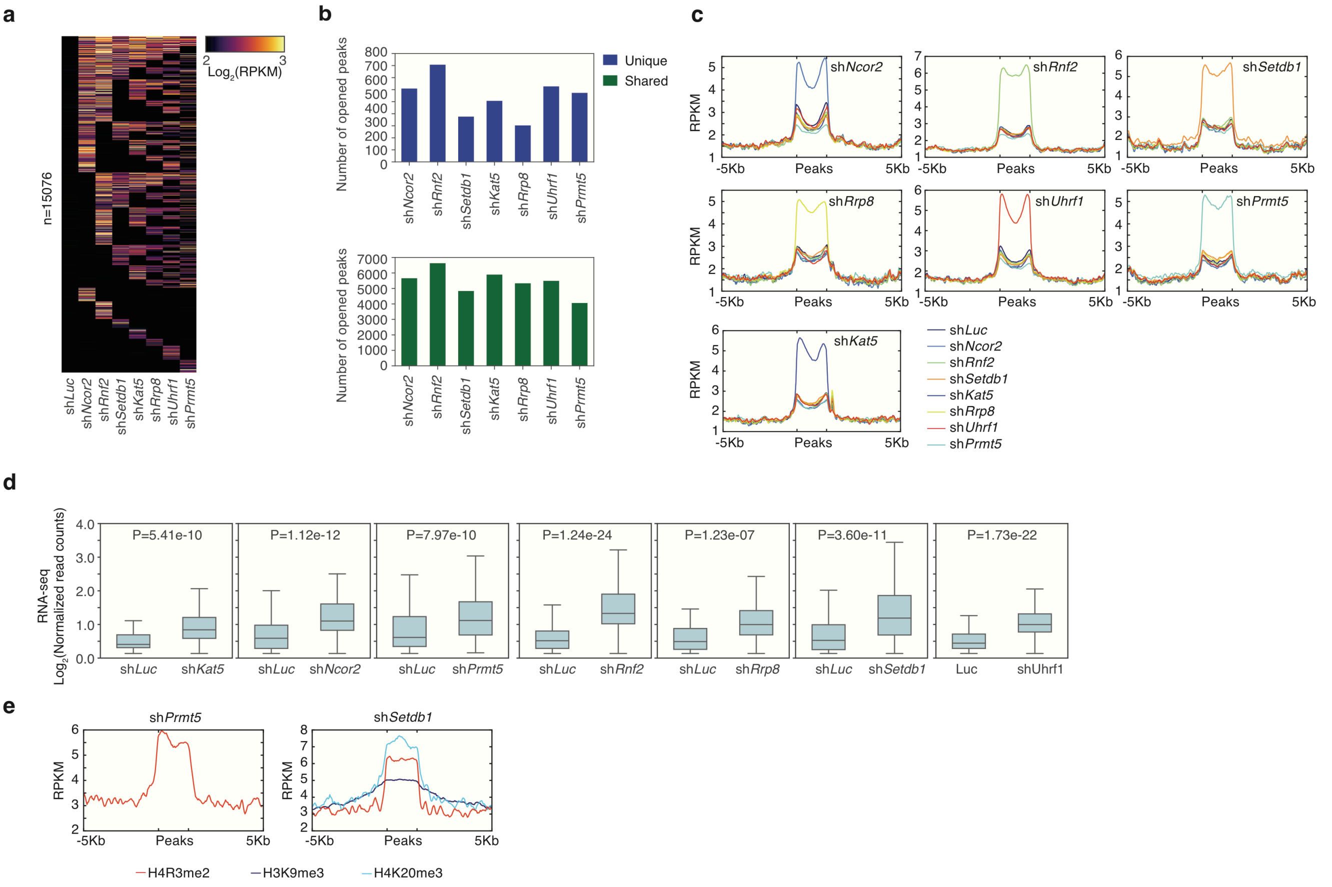

Supplementary Figure 6. Loss of CMs leads to CM-specific opening of chromatin. (a) All ATAC peaks that change from closed to open in any knockdown. A peak was considered open if it had an average RPKM>4, and closed with an RPKM<4. (b) Number of individual peaks that become open in an shRNA knockdown that was unique, or shared in any 2 shRNA knockdowns. (c) Pileup tag counts of ATAC-seq data across those peaks that become accessible in the indicated CM knockdown. The peaks were scaled to the same size, and the flanking 5kb regions are shown. (d) ATAC-seq peaks that gain accessible chromatin after the indicated shRNA knockdown, have significantly up-regulated expression of the corresponding flanking genes. The expression of all genes that were within 10kb of the opened peak in the ATAC-seq data was measured. Comparisons are between the sh*Luc* control and the indicated CM knockdown. Significance was derived from a Mann-Whitney U test. For the boxplots, the midline indicates the median, boxes indicate the upper and lower quartiles and the whiskers indicate 1.5*interquartile range. (e) ATAC peaks that become open in the indicated shRNA knockdown, are enriched for the corresponding chromatin mark. Examples are shown for sh*Prmt5* (H3R3me2 catalytic enzyme), and sh*Setdb1* (H3K9me3 catalytic enzyme).

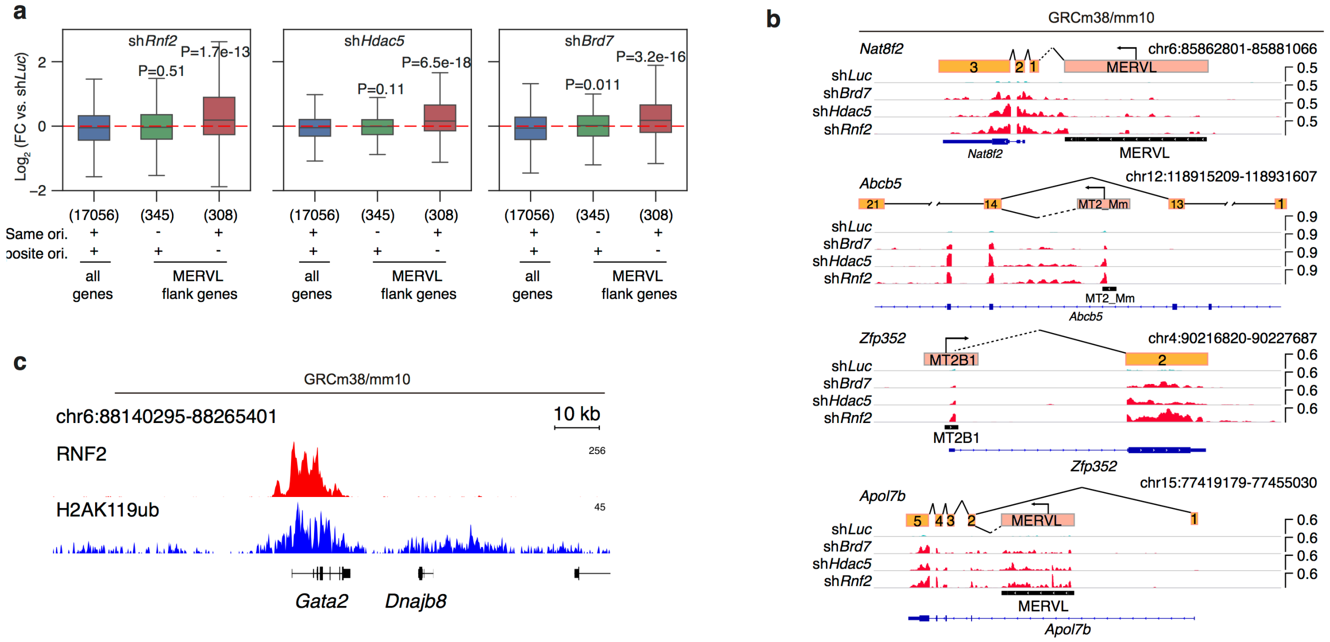

Supplementary Figure 7. Strand-specific splicing of MERVLs and MT2_Mm into 2C-related genes, and RNF2 and H2AK119ub labelling of the *Gata2* locus in ESCs. (a) Fold-change expression level versus sh*Luc* for all genes, and those genes that have a flanking or intronic MERVL on the same orientation (same ori.) or opposite orientation (opposite ori.) as the gene. All genes are shown for comparison. Significance is derived from a Mann-Whitney U test, compared to all genes. For the boxplots, the midline indicates the median, boxes indicate the upper and lower quartiles and the whiskers indicate 1.5*interquartile range. (b) Example genome views of alternatively spliced transcripts that splice a MERVL or MERVL-related TE (MT2, MT2B1) exon into a splice isoform. *Zfp352* and *Abcb5* were from[13]. (c) Genome view of RNF2 binding and H2AK119ub levels at the *Gata2* locus in ESCs. ChIP-seq data was taken from GSE76825[14].

Figure 3c

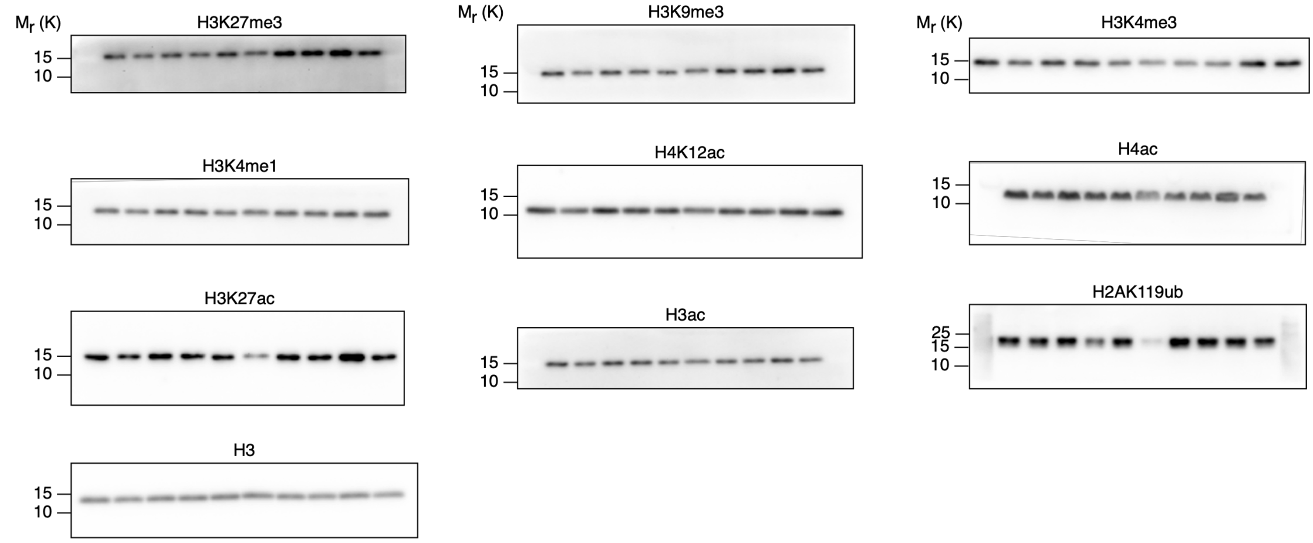

Supplementary Figure 5c

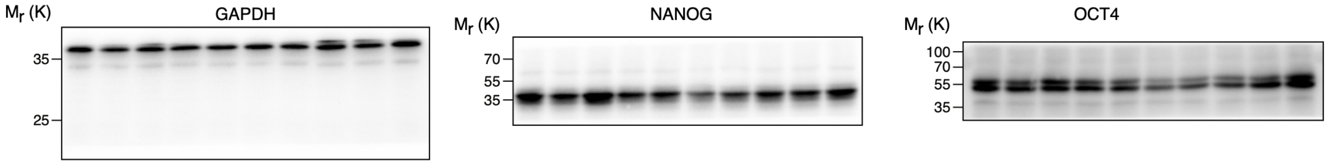

Supplementary Figure 8. Raw images of Western blots. Top panel shows the Westerns for **Figure 3c** and the bottom panels show the Western blots for **Supplementary Figure 5c**. The black borders indicate the boundaries of the blotting membrane. The molecular weight markers are indicated with Mr (K).

Supplementary References

1 Tan, M. *et al.* Identification of 67 histone marks and histone lysine crotonylation as a new type of histone modification. *Cell* **146**, 1016-1028, doi:10.1016/j.cell.2011.08.008 (2011).

2 Arnaudo, A. M. & Garcia, B. A. Proteomic characterization of novel histone post-translational modifications. *Epigenetics & chromatin* **6**, 24, doi:10.1186/1756-8935-6-24 (2013).

3 Sadakierska-Chudy, A. & Filip, M. A comprehensive view of the epigenetic landscape. Part II: Histone post-translational modification, nucleosome level, and chromatin regulation by ncRNAs. *Neurotoxicity research* **27**, 172-197, doi:10.1007/s12640-014-9508-6 (2015).

4 Dai, L. *et al.* Lysine 2-hydroxyisobutyrylation is a widely distributed active histone mark. *Nature chemical biology* **10**, 365-370, doi:10.1038/nchembio.1497 (2014).

5 Kebede, A. F., Schneider, R. & Daujat, S. Novel types and sites of histone modifications emerge as players in the transcriptional regulation contest. *The FEBS journal* **282**, 1658-1674, doi:10.1111/febs.13047 (2015).

6 Wheeler, D. L. *et al.* Database resources of the National Center for Biotechnology Information. *Nucleic acids research* **36**, D13-21, doi:10.1093/nar/gkm1000 (2008).

7 Bailey, T. L. *et al.* MEME SUITE: tools for motif discovery and searching. *Nucleic acids research* **37**, W202-208, doi:10.1093/nar/gkp335 (2009).

8 Heng, J. C. *et al.* The nuclear receptor Nr5a2 can replace Oct4 in the reprogramming of murine somatic cells to pluripotent cells. *Cell Stem Cell* **6**, 167-174, doi:10.1016/j.stem.2009.12.009 (2010).

9 Bao, S. *et al.* Derivation of hypermethylated pluripotent embryonic stem cells with high potency. *Cell research* **28**, 22-34, doi:10.1038/cr.2017.134 (2018).

10 Pastor, W. A. *et al.* TFAP2C regulates transcription in human naive pluripotency by opening enhancers. *Nat Cell Biol* **20**, 553-564, doi:10.1038/s41556-018-0089-0 (2018).

11 Medvedeva, Y. A. *et al.* EpiFactors: a comprehensive database of human epigenetic factors and complexes. *Database : the journal of biological databases and curation* **2015**, bav067, doi:10.1093/database/bav067 (2015).

12 Hutchins, A. P. *et al.* Models of global gene expression define major domains of cell type and tissue identity. *Nucleic acids research* **45**, 2354-2367, doi:10.1093/nar/gkx054 (2017).

13 Choi, Y. J. *et al.* Deficiency of microRNA miR-34a expands cell fate potential in pluripotent stem cells. *Science* **355**, doi:10.1126/science.aag1927 (2017).

14 Li, H. *et al.* RNA Helicase DDX5 Inhibits Reprogramming to Pluripotency by miRNA-Based Repression of RYBP and its PRC1-Dependent and -Independent Functions. *Cell Stem Cell* **20**, 462-477.e466, doi:10.1016/j.stem.2016.12.002 (2017).
