# Supplementary material for: Transposable elements are regulated by context-specific patterns of chromatin marks in mouse embryonic stem cells
Source: Nat Commun. 2019 Jan 3;10:34. doi: 10.1038/s41467-018-08006-y (PMC6318327; doi:10.1038/s41467-018-08006-y)
Supplement: Supplementary file 2 — Description of Additional Supplementary Files [file 41467_2018_8006_MOESM2_ESM.pdf]

Details of Supplementary Data files

Supplementary Data 1. Details of the ChIP-seq, RNA-seq and ATAC-seq experiments used in this study, including mapping statistics for all libraries generated

Supplementary Data 2. Log2 fold enrichment of chromatin modifications for selected and all TE types

Supplementary Data 3. Table containing all and a selected list of TE types and their log2 fold-enrichment of bound CMs

Supplementary Data 4. Table containing all expressed TE types, and their expression level (normalized tag count) in response shRNA knockdown (RNA-seq)

Supplementary Data 5. Table containing all types of TE, and their normalized chromatin expression level (in RPKM), and fold-change in response to shRNA knockdown (ATAC-seq)

Supplementary Data 6. shRNA sequences used for the knockdown experiments

Supplementary Data 7. Primer sequences for TE RT-qPCR and ChIP-qPCR

Supplementary Data 8. Source Data File. File containing the raw numerical data from Figure 1d, 3h and Supplementary Figures 2h and 5b.
